# Biomechanical changes in the gastrocnemius medius–Achilles tendon complex in people with hypermobility spectrum disorders: A cross-sectional compression sonoelastography study

**DOI:** 10.3389/fmed.2023.1062808

**Published:** 2023-01-19

**Authors:** Najla Alsiri, Shea Palmer

**Affiliations:** ^1^Al-Razi Orthopedics and Rehabilitation Hospital, Kuwait, Kuwait; ^2^Centre for Care Excellence, University Hospitals Coventry and Warwickshire NHS Trust, Coventry University, Coventry, United Kingdom

**Keywords:** connective tissue, hypermobility, Ehlers-Danlos, soft, ultrasound, sonoelastography

## Abstract

**Objective:**

This study aimed to assess the biomechanical impact of Hypermobility Spectrum Disorders (HSD) on the elasticity of the gastrocnemius medius-Achilles tendon (GM-AT) complex.

**Methods:**

Using a cross-sectional design, the GM-AT complex elasticity was compared using sonoelastography (SEG) in an HSD group and healthy controls during rest and maximal isometric plantar flexion contraction.

**Results:**

The HSD group comprised 28 patients (26 women); mean ± SD age 28.7 ± 8.4 years, compared to 28 controls (26 women); 31.5 ± 8.7 years. During rest, greater elasticity was identified in HSD relative to controls at the GM-AT musculotendinous junction (strain ratio 2.05 ± 1.31 vs. 1.48 ± 0.49), mid-AT (3.60 ± 1.97 vs. 2.66 ± 1.00), and distal AT (4.57 ± 2.69 vs. 3.22 ± 1.94) (all *p* < 0.05). During contraction, no significant differences were found between groups at the GM-AT musculotendinous junction (3.40 ± 2.16 vs. 2.62 ± 1.07), mid AT (10.75 ± 5.29 vs. 8.49 ± 3.53), or distal AT (8.55 ± 5.39 vs. 8.83 ± 3.51) (all *p* > 0.05). No significant differences were found between groups in the GM strain ratio during rest (4.05 ± 1.43 vs. 3.62 ± 0.78), or contraction (4.23 ± 1.29 vs. 4.19 ± 1.31). Exploratory Receiver Operator Characteristics curve analysis suggested low sensitivity and specificity of the strain ratio for the diagnosis of HSD.

**Conclusion:**

People with HSD have greater GM-AT complex elasticity. Although statistically significant group differences were identified, further research is required to establish the diagnostic, clinical, and research utility of strain ratio measurements.

## 1. Introduction

Hypermobility Spectrum disorders (HSD) are connective tissue disorders characterized by symptomatic synovial joint hypermobility etiologically related to genetic and pathologic factors (1–5). HSD is a new terminology introduced in 2017 for hypermobility-related disorders to highlight the wide heterogeneities within joint hypermobility-related conditions, and to replace the terms “Joint Hypermobility Syndrome” (JHS) and “Ehlers-Danlos Syndrome Hypermobility Type” (EDS-HT) (6). HSD diagnosis is likely to capture the majority of patients previously diagnosed as JHS/EDS-HT (7). The subdivisions of HSD are localized, peripheral, generalized, and historical HSD (6). HSD is the most common type of connective tissue disorder, where its prevalence was 55% of women patients attending physiotherapy with musculoskeletal symptoms (8). The prominent symptomatic features of HSD include chronic symptomatic joint hypermobility, recurrent joint dislocation, and muscle weakness (2, 9–14). It is a multi-systemic disorder, affecting the musculoskeletal, cardiovascular, digestive, and autonomic nervous systems, as it alters the mechanical properties of these systems’ connective tissues (2).

Hypermobility spectrum disorders lacks a consistent and fixed constitutional pattern as it involves huge spectra of symptomatic features, severity levels, and systemic involvement, which complicate its identification. The complexity of HSD identification and diagnosis is highly related to the subjectivity of the approaches used, as they rely on past medical history and clinical examination using the historical Brighton or Villefranche criteria or the 2017 HSD classification framework (2, 6, 7, 15, 16). The current approaches are highly dependent on the experience of the examiner, and the memory of the patient. The predominant clinical feature of joint hypermobility, assessed using the Beighton score, is used to guide the identification process, but could be affected by the regression of the condition, and replaced with joint stiffness, which further complicates the diagnostic process. A recent review related the diagnostic delay of hEDS/HSD to the lack of a confirmatory diagnostic approach (17). Ultimately, there is a need for a more objective diagnostic procedure to improve the subjectivity of existing diagnostic approaches.

Advances in radiology medicine could offer a more objective approach for the diagnosis of HSD. Compression sonoelastography (SEG) is an ultrasound-based system which is designed to examine the biomechanical properties of structures through color-coded images and strain ratio (18, 19). The strain ratio is an objective elasticity measure provided in real-time (18, 19). Introducing SEG to assist in the diagnosis of HSD could be valuable as a more objective indicator. SEG may be able to detect alterations in the biomechanical properties of the musculotendinous structures in HSD theoretically caused by several factors. Firstly, the biomechanical rigidity of musculotendinous structures in HSD could be reduced due to genetic mutations in the gene encoding collagen, which is essential to confer tensile strength and rigidity (4, 10, 20). Biomechanical properties could also be altered as a result of the primary features of HSD, including joint hypermobility and muscular weakness (2, 4, 10). Such genetic and symptomatic factors strongly suggest changes in the biomechanical properties of the musculotendinous structures in HSD. Consequently, SEG could help in HSD identification, diagnosis, and monitoring.

The value of SEG for the examination of people with HSD was previously explored by our research group in two studies (21, 22). The first study supported the feasibility of SEG in terms of training requirements, patient tolerance, and examination of the GM muscle in JHS (21). The second study found significant reductions in musculoskeletal elasticity in people with HSD when compared to a control group in various anatomical structures (22). That study provided initial evaluations for the deltoid muscle, biceps brachii muscles, brachioradialis muscle, rectus femoris muscle, patellar tendon, and the gastrocnemius-Achilles tendon (GM-AT) complex, but none were examined intensively (22). However, it was clear that the GM-AT complex showed the highest differences between groups and the least error rate in comparison to the other structures examined (22). Alsiri et al. (22) strongly suggested intensively exploring the GM-AT complex in people with HSD relative to a control group as a promising indicator for identifying, diagnosing, and monitoring changes in biomechanical properties in response to the progression of the condition, or to interventions (22).

The current intensive study of the GM-AT complex was therefore conducted to provide valuable clinical indications and recommendations for clinical examination, for example specifying the exact anatomical region and muscle contraction status to be used. To explore the clinical value of SEG examinations, the predominant symptomatic features of HSD were explored and correlated with HSD findings, including joint hypermobility and pain. We hypothesize that HSD has a significant impact in reducing the stiffness of the GM-AT complex. The study aimed to explore the biomechanical properties of the GM-AT complex in people with HSD through comparison with a control group using compression SEG at different anatomical regions and during rest and maximal isometric contraction. Secondary aims were to correlate SEG findings with the major symptomatic features of HSD including joint hypermobility and pain to determine the clinical value of SEG results, and to undertake exploratory Receiver Operating Characteristics (ROC) curve analysis to explore potential diagnostic performance.

## 2. Materials and methods

### 2.1. Study design, ethics, and funding

European Alliance of Associations for Rheumatology (EULAR) recommendations for the reporting of ultrasound studies in rheumatic and musculoskeletal diseases (23) and The Strengthening the Reporting of Observational Studies in Epidemiology (STROBE) publication standards for cross-sectional studies were followed (24). A cross-sectional prospective between-group research design was employed, and to ensure between-group homogeneity, a matching pairs design was used in terms of age and sex (25). The study was approved by the Kuwait Ministry of Health (2018/923) in accordance with the declaration of Helsinki, where written informed consent was obtained, and voluntary participation and confidentiality were maintained. SEG is a safe procedure with minimal radiation hazards. The Kuwait Foundation for the Advancement of Sciences funded the study (PR19-13NP-01).

### 2.2. Inclusion and exclusion criterion

Female and male subjects aged ≥ 18 years old were invited to take part in the study. Patients were included in the HSD group if they met the 2017 HSD criteria (6). Participants from both groups were excluded if they had a recent injury within the last 3 months in the lower limbs; had recent surgery of the lower limbs during the last 12 months, or were pregnant or gave birth during the last year, due to postpartum ligament laxity (26). Exclusion criteria for the control group were: generalized joint laxity (≥ 4/9 in the Beighton score); recent pain within the last 3 months in the lower limb joints; or any condition which causes weakness to the lower limbs.

### 2.3. Sample size determination

Sample size calculation was based on the only previous investigation of the impact of HSD on AT elasticity using compression SEG (22). Using the mean (standard deviation) of the higher boundary of strain index for the AT of 0.24 (0.06) for the HSD group, and 0.49 (0.13) for the control group (*p* = 0.001), an effect size of 0.77 was identified. The required sample size was estimated to be 28 participants per group for a two tailed hypothesis at an α-level of 0.05 and a power (1 − ß) of 0.8.

### 2.4. Recruitment sites and strategy

Patients with HSD were recruited from Al-Razi Orthopedic Hospital, Kuwait, and healthy participants were recruited from hospital staff and their relatives who met the eligibility criteria. The recruitment site serves the entire population of Kuwait and is therefore likely to have generated a representative sample. Regarding the HSD group, recruitment packs (containing an invitation letter, participant information sheet, and reply slip) were prepared and distributed in the physiotherapy department and in orthopedic clinics. People willing to participate contacted the chief investigator to check the eligibility criteria and book an examination appointment. Sixteen participants were examined between November 2019 and February 2020. The study paused during the COVID-19 pandemic before resuming from November 2021 to March 2022.

### 2.5. Instrumentation

**FIGURE 1:**
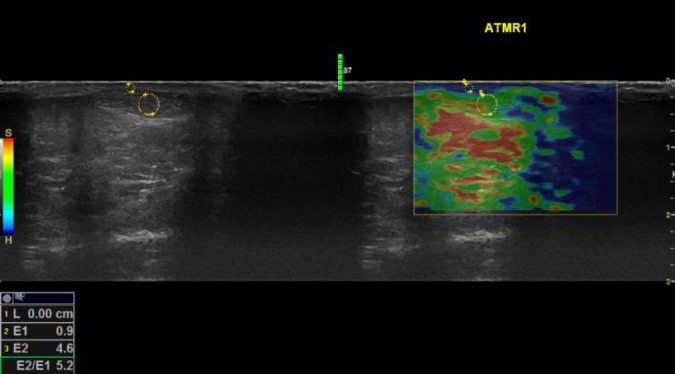
Sonoelastography images of the mid Achilles tendon examined during rest for a participant with hypermobility spectrum disorder. Keys: Red refers to soft, green refers to medium, and blue refers to hard tissue. E2/E1 refers to the strain ratio; the higher the strain ratio, the higher the elasticity (41). Words in yellow are coding written by the operator for saving purposes. Four compressions were applied for each examination, and an image associated with the third compression was selected to attain the strain ratio. Using the colored SEG image, the position of the image box was moved to incorporate the AOI with an adjacent subcutaneous fat layer as a reference. A circle was traced to encompass the entire mid portion of the AOI, then another circle was traced to encompass an adjacent subcutaneous fat layer as a reference encoding blue color pixels. The size of the traced area as a reference has no influence on the strain ratio (42). The two areas were selected to be as adjacent as possible to ensure that they were subjected to the same magnitude of compressions. Musculoskeletal structures lack fat tissues; therefore, subcutaneous fat is the most applicable for musculoskeletal examination, which shows comparable reliability compared to using alternative areas (42).

Compression SEG was used to assess the elasticity of the GM-AT complex (LOGIQ S7 Expert; ORTNER Medizintechnik e.U), with a 6–15 MHz linear-array high-frequency transducer. SEG is an ultrasonography-based system designed to assess tissue elasticity, by measuring the perpendicular displacement and strain of the examined tissues in response to the examiner’s external mild compressions applied using the ultrasound transducer (18, 19, 27). SEG is equipped with a visual indicator to ensure the quality of the compressions applied by the examiner (27). Tissue displacement is calculated and converted in real time into color-coded images which reflect the elasticity of the examined tissues; red refers to soft, green refers to medium, and blue refers to hard tissues (19, 27) (Figure 1). The scoring system for the SEG is the strain ratio, which is a semi-quantitative stiffness measure obtained in real time by comparing the strain index of the area of interest (AOI), in this case the GM-AT complex, relative to the strain index of an adjacent fat layer. SEG is quick, practical and reliable for examining the biomechanical properties of the musculoskeletal system, including the GM-AT complex (19, 22, 28). VASs were used to assess the average pain intensity at the lower limb joints, bilaterally. VAS questions were limited to pain experienced during the past week to eliminate errors arising from recall. VAS is simple, sensitive, valid, and has high test-retest reliability ranging from 0.97 to 0.99 (29–32).

### 2.6. Data collection procedures

Procedural acquisitions and readings were performed at the same time by the same operator (N.A). The operator has a doctorate in orthopedic rehabilitation, has received postgraduate training in musculoskeletal ultrasound and has 7 years’ experience in sonoelastography and musculoskeletal ultrasound. The reliability of the operator was previously established for SEG examination of the GM-AT complex, with ICC values of 0.893 and 0.824, for the GM and the AT, respectively (22). Three days of piloting took place before study data collection. Eligibility criteria were checked by the operator before examination, so assessment was not blinded.

During the examination appointment, participants were asked to read and sign the consent form, then the diagnosis of HSD was confirmed using the 2017 classification framework (7). The inclusion and exclusion criteria were checked, then the participant’s height and weight were measured. The demographic data sheet was filled, and participants were asked to answer the VASs for pain.

For SEG examination, GM-AT complex elasticity was considered as the domain component. The operational definition of the target domain component was the higher the strain ratio, the higher the elasticity of the tissues in the HSD group in comparison with the control group; with the corresponding theoretical definition being that soft tissue deforms more than hard tissue in response to the operator’s dynamic mild compressions applied during examination. B-mode ultrasound was used first to identify the AOI, and then SEG was activated to examine the biomechanical properties of the posterior surface of the GM-AT complex. The same SEG device was used for the entire study, and the settings for musculoskeletal examination were used in accordance with the guidelines of the European Federation of Societies for Ultrasound in Medicine and Biology (EFSUMB) (33). The duration of the SEG examination was 30–35 min, room temperature was maintained at 22°C, and the examinations took place between 10 a.m. and 1 p.m. Participants were instructed not to undertake exercise prior to examination of 48 h, and all reported that they had complied with this instruction.

The mechanical properties of the GM-AT complex were examined during: (1) rest, and (2) maximal isometric plantar flexion contraction. During each contraction status, the GM-AT complex was examined at four points: (1) proximal 30% length of the GM, determined by calculating the proximal 30% length from the midpoint of the popliteal fossa to the midpoint between the ankle malleoli; (2) GM-AT musculotendinous junction determined with B-mode ultrasound; (3) mid AT, determined as the midpoint between the two malleoli; and (4) distal AT, near the AT insertion into the calcaneus (34). The most practical and repeatable planes for examination were used—longitudinal for the GM, and transverse for the GM-AT musculotendinous junction, mid AT, and distal AT. Each examination was performed twice, and the mean values were used for analysis. The participants were asked to lie in a prone position while keeping the knee in full extension. For the examination at rest, the slack length was used, where the feet were hung over the edge of the examination plinth in a comfortable ankle joint position.

### 2.7. Statistical analysis

Following a pre-specified statistical analysis plan, Statistical Package for the Social Sciences (SPSS) was used for data analysis (IBM SPSS Statistics for Windows, Version 23.0. Armonk, NY, USA, IBM). Means and standard deviations were used as descriptive statistics. Shapiro–Wilk tests were used to assess the normal distribution of the data. The majority of the data were normally distributed; therefore, the parametric option of independent samples *t*-tests were employed for between-group comparisons. A *p*-value of ≤0.05 was defined as statistically significant (35). Pearson Product correlation coefficients were used to assess interrelationships between variables. There were no missing data. Where statistically significant differences in strain ratio were found between the two groups, exploratory ROC curve analysis was undertaken to explore potential diagnostic performance.

## 3. Results

The HSD group included 28 patients, 26 women and two men, aged 28.7 ± 8.4 years (mean ± SD). The control group included 28 participants, 26 women and two men, aged 31.5 ± 8.7 years. No statistically significant differences were found between the two groups for age, height, or weight (all *p* > 0.05), which indicate that the two groups are homogenous at baseline (Table 1). The Beighton score was significantly higher in the HSD group (*p* = 0.001) (Table 1). The HSD group included 20 patients with generalized HSD, seven patients with localized HSD, and one patient with historical HSD. Three patients from the HSD group, and one participant from the control group were left side dominant. In terms of ethnicity, all participants in both groups were Asian. Nationalities of women in the HSD group included Kuwait (*n* = 22), Saudi Arabia (*n* = 1), Philippines (*n* = 1), India (*n* = 1), and Lebanon (*n* = 1). Men in the HSD group were from Kuwait (n = 1) and Egypt (*n* = 1). Women in the control group were from Kuwait (*n* = 15), India (*n* = 9), Egypt (*n* = 1), and Syria (*n* = 1). Men in the control group were from Kuwait (*n* = 1) and Saudi Arabia (*n* = 1).

**TABLE 1:** The demographic characteristics of the hypermobility spectrum disorders (HSD) group compared to the control group.

Demographic characteristics	HSD group (*n* = 28)	Control group (*n* = 28)	*P*-value
	Mean ± standard deviation	Mean ± standard deviation	
Age (years)	28.7 ± 8.4	31.5 ± 8.7	0.23
Height (cm)	162.5 ± 7.3	160.5 ± 8.5	0.35
Weight (kg)	68.3 ± 13.0	67.4 ± 12.0	0.79
Beighton score	4.53 ± 1.91	0.53 ± 0.83	0.001*

*Indicates statistically significant difference at *p*-value of ≤0.05 by independent sample *t*-test.

During rest, statistically significantly greater elasticity was identified in the HSD group relative to the control group at the GM-AT musculotendinous junction (strain ratio 2.05 ± 1.31 vs. 1.48 ± 0.49, respectively), mid AT (3.60 ± 1.97 vs. 2.66 ± 1.00), and distal AT (4.57 ± 2.69 vs. 3.22 ± 1.94) (all *p* < 0.05) (Table 2). During maximal isometric plantar flexion contraction, the descriptive statistics showed higher elasticity in the HSD group compared to the control group in GM-AT musculotendinous junction (3.40 ± 2.16 vs. 2.62 ± 1.07), and mid AT (10.75 ± 5.29 vs. 8.49 ± 3.53). However, the observed differences were not statistically significant (all *p* > 0.05) (Table 2). The descriptive statistics pointed toward higher elasticity of the GM in HSD relative to controls during rest (4.05 ± 1.43 vs. 3.62 ± 0.78), but not during contraction (4.23 ± 1.29 vs. 4.19 ± 1.31) and neither were statistically significant (both *p* > 0.05) (Table 2). No significant differences were found between the two groups in the cross-sectional areas (all *p* > 0.05) (Table 2).

**TABLE 2:** Biomechanical and morphological comparison between the hypermobility spectrum disorders (HSD) group and the control group measured with compression sonoelastography and B-mode ultrasound, respectively.

	HSD group (*n* = 28)	Control group (*n* = 28)	*P*-value	Mean difference	95% CI
	Mean ± standard deviation	Mean ± standard deviation			
**Biomechanical comparison of strain ratio measured during rest**					
GM	4.05 ± 1.43	3.62 ± 0.78	0.169	0.43	−0.18, 1.05
GM-AT musculotendinous junction	2.05 ± 1.31	1.48 ± 0.49	0.036*	0.57	0.03, 1.10
Mid AT	3.60 ± 1.97	2.66 ± 1.00	0.028*	0.94	0.10, 1.78
Distal AT	4.57 ± 2.69	3.22 ± 1.94	0.037*	1.34	0.08, 2.60
**Biomechanical comparison of strain ratio measured during maximal isometric planter flexion contraction**					
GM	4.23 ± 1.29	4.19 ± 1.31	0.911	0.03	−0.65, 0.73
GM-AT musculotendinous junction	3.40 ± 2.16	2.62 ± 1.07	0.092	0.78	−0.13, 1.69
Mid AT	10.75 ± 5.29	8.49 ± 3.53	0.065	2.26	−0.14, 4.67
Distal AT	8.55 ± 5.39	8.83 ± 3.51	0.824	−0.27	−2.70, 2.16
**Morphological comparison of GM–AT complex cross-sectional area (cm)**					
GM	1.42 ± 0.34	1.49 ± 0.35	0.430	−0.07	−0.26, 0.11
GM-AT musculotendinous junction	0.34 ± 0.17	0.35 ± 0.16	0.875	−0.00	−0.10, 0.08
Mid AT	0.41 ± 0.09	0.38 ± 0.08	0.285	0.02	−0.02, 0.07
Distal AT	0.31 ± 0.12	0.40 ± 0.40	0.324	−0.08	−0.25, 0.08

The higher the strain ratio, the higher the elasticity of the examined structure (37). GM, gastrocnemius medius muscle, AT, Achilles tendon, CI, confidence interval.

*Indicates a statistically significant difference at *p*-value of ≤0.05 by independent sample *t*-test.

The intensity of joint pain was significantly greater among the HSD group at the hip, knee, and ankle joints, bilaterally (all *p* < 0.001) (Table 3). During rest, weak but statistically significant correlations were found between the intensity of hip joint pain and the strain ratio of the GM; *r* = 0.362 (*p* = 0.008), and between the intensity of knee joint pain and the strain ratio of the GM-AT musculotendinous junction; *r* = 0.360 (*p* = 0.008) (Table 4). During maximal contraction, weak but statistically significant correlations were determined between the Beighton score and the strain ratio of the GM-AT musculotendinous junction and mid AT; *r* = 0.318 (*p* = 0.017) and *r* = 0.267 (*p* = 0.047), respectively (Table 4). The Beighton score showed significant correlations with pain intensity at the hip, knee and ankle joints (all *p* < 0.05) (Table 4).

**TABLE 3:** The intensity of joint pain (measured with visual analogue scales) for the hypermobility spectrum disorders (HSD) group compared to the control group.

	HSD group (*n* = 28)	Control group (*n* = 28)	*P*-value	Mean difference	95% CI
	Mean ± standard deviation	Mean ± standard deviation			
**Dominant side**					
Hip joint	2.04 ± (3.43)	0.09 ± (0.51)	0.005*	1.95	0.63, 3.26
Knee joint	2.46 ± (2.89)	0.00 ± (0.00)	0.001*	2.46	1.36, 3.55
Ankle joint	2.74 ± (3.40)	0.00 ± (0.00)	0.001*	2.74	1.45, 4.02
**Non-dominant side**					
Hip joint	2.40 ± (3.24)	0.00 ± (0.00)	0.001*	2.40	1.17, 3.62
Knee joint	2.87 ± (3.03)	0.27 ± (0.83)	0.001*	2.87	1.72, 4.02
Ankle joint	3.59 ± (3.79)	0.00 ± (0.00)	0.001*	3.95	2.15. 5.03

*Indicates a statistically significant difference at <0.05 by independent sample *t*-test.

**TABLE 4:** The relationship between the intensity of joint pain (measured with visual analogue scales), Beighton score, and the strain ratio (measured with compression sonoelastography of the GM-AT complex).

	Relationship statistics	Beighton score	Hip pain	Knee pain	Ankle pain
**Strain ratio measured during rest**					
GM	*r*	0.202	0.362*	0.144	0.195
GM-AT musculotendinous junction	*p*-value	0.135	0.008*	0.416	0.161
	*r*	0.101	0.045	0.360*	0.144
Mid AT	*p*-value	0.458	0.748	0.008*	0.303
	*r*	0.122	0.160	0.262	0.047
Distal AT	*p*-value	0.370	0.252	0.058	0.738
	*r*	0.220	0.182	0.055	0.097
	*p*-value	0.103	0.193	0.696	0.489
**Strain ratio measured during maximal isometric planter flexion contraction**					
GM	*r*	0.029	0.104	0.001	0.112
GM-AT musculotendinous junction	*p*-value	0.832	0.460	0.997	0.426
	*r*	0.318*	0.214	0.216	0.089
Mid AT	*p*-value	0.017*	0.124	0.120	0.525
	*r*	0.267*	0.144	0.172	0.128
Distal AT	*p*-value	0.047*	0.305	0.218	0.360
		*r*	0.045	−0.042	−0.024	−0.071
	*p*-value	0.744	0.767	0.862	0.613
Beighton score		*r*	0.472*	0.568*	0.637*
		*p*-value	0.001*	0.001*	0.001*

GM, gastrocnemius medius; AT, Achilles tendon. Joint pain intensity presents the dominant side, where a sonoelastography examination was performed.

*Statistically significant correlation (2-tailed).

**FIGURE 2:**
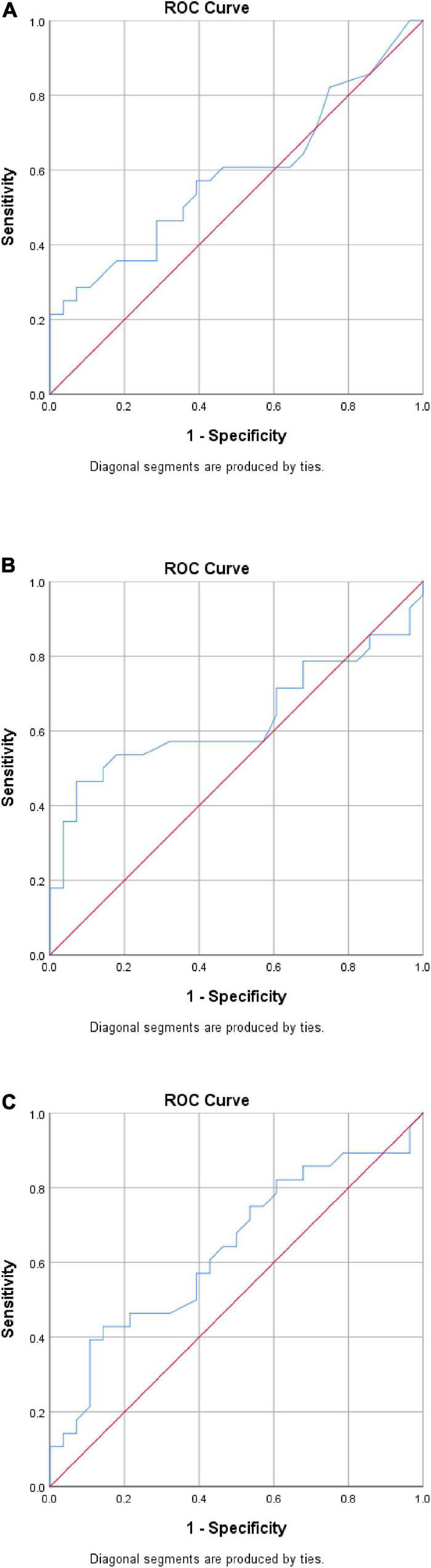
Exploratory receiver operating characteristic (ROC) curve analysis. **(A)** Proximal AT. AUC = 0.589 (95% CI 0.437, 0.740), *p* = 0.255. **(B)** Mid-AT. AUC = 0.631 (95% CI 0.477, 0.784), *p* = 0.093. **(C)** Distal AT. AUC = 0.635 (95% CI 0.488, 0.782), *p* = 0.082. CI, confidence interval; AUC, analysis of the area under the curve.

Receiver operating characteristics curves for the strain ratio at the proximal, mid, and distal AT at rest are presented in Figure 2. These suggested that the strain ratio may not, on its own, distinguish between those with and without HSD. This was confirmed by analysis of the area under the curve (AUC), yielding a maximum point estimate of 0.635 for the distal AT (Figure 2). None of the AUC estimates were statistically significant (all *p* > 0.05). Linear regression equations were constructed to predict the strain index, but the R square values were very low (Appendix).

## 4. Discussion

This is the first study to intensively explore the GM-AT complex in people with HSD using SEG technology. The findings from this study have implications for presenting an objective procedure to assist in the clinical examination of patients with HSD in terms of the diagnosis, identification process and monitoring of progression. Statistically significant reductions in the strain ratio were identified in the HSD group relative to the control group during rest at the musculotendinous junction, mid AT, and distal AT. Strain ratio was also found to be reduced during maximal isometric plantar flexion contraction, yet the reductions observed did not reach statistical significance. The objective procedure introduced shows that using SEG might be clinically valuable as it demonstrated weak but significant correlations with the major symptomatic features of HSD, including joint hypermobility and pain intensity at the lower limb joints. Although there were statistically significant between-group differences at the proximal, mid and distal AT strain ratio at rest, ROC curve analysis suggested that the strain ratio may not have adequate sensitivity and specificity when used alone as a diagnostic criterion. SEG might need to be combined with clinical examination to enhance its diagnostic capability. However, it should be noted that the study was not specifically designed to explore sensitivity and specificity and is likely to have been under-powered for such analysis (some *p*-values were approaching statistical significance). Further specific investigation of strain ratio as a potential diagnostic criterion is warranted, in both HSD and hEDS.

Various elements related to etiological and symptomatic features could explain the reduction in strain ratio observed the GM-AT complex in HSDs. The biomechanical properties of the musculotendinous structures are highly reliant on the connective tissues. Specifically, the connective tissues bind the underlying structures and form a framework of support to maintain the strength of the musculoskeletal system. Indeed, HSDs are connective tissue disorders, and collagen is the main type of fibers found in the connective tissues (36). Collagen gives the musculoskeletal structures their mechanical strength, and forms 65–80% of tendon composition (36). However, various studies have found mutations, and molecular defects in the genes encoding collagen, and in the enzymes responsible for collagen modification in people with JHS/EDS-HT (1, 4). However, the genetic etiology of HSD is not yet conclusive and is sometimes contradictory. The etiology has also been related to abnormalities in an extracellular glycoprotein (tenascin-X), which bridges between collagen fibers and contributes toward collagen formation, and in fibroblast mechanobiology of cell adhesion and cytoskeleton organization (20, 37). The abnormalities of vital components of the connective tissues in HSD could explain the significant reduction in the strain ratio of the GM-AT complex identified by the current study. Moreover, muscle weakness in HSD could explain the reduced strain ratio. Strength was found to be significantly reduced in HSD including the muscles of the shoulder, hand, hip, knee, and foot (15, 26, 38). Muscle strength was associated with activity limitation in HSD, which could lead to a vicious cycle of further weakness due to the muscular disuse effect (15). Muscle strength could be further reduced due to participation restrictions due to joint pain in HSD, increasing the impact of the symptomatic features on reducing the strain ratio of musculotendinous structures (21).

Two studies were performed previously using SEG to explore the GM-AT complex in HSDs during rest (21, 22), but contraction status was not explored previously. The midpoint of the AT was explored at rest in HSD by one previous study (22). The mean difference between the two groups of the mid-AT was relatively similar at 1.32 in the previous study (22), and 0.94 in the current study. However, no significant difference in the strain ratio was identified by the previous study, while the current study found increased elasticity of the AT at the mid-point. The current study was designed to be more powerful in terms of sample size for AT examination, which could explain the ability of the current study to detect between-group differences. The previous study included 21 patients with HSD, and so the sample size increased by 33.3% in the current study, reaching 28 patients in the HSD group (22). The GM muscle was examined in both previous studies, supporting the current identification of the insignificant impact of HSD on the GM biomechanical properties (21, 22). The first was a small feasibility study comparing ten patients with JHS relative to ten healthy controls (21). However, the findings cannot be directly compared because of using different analysis approaches, where software was used to count the color pixels of the colored SEG images in the previous study (21). Even so, no significant differences were found in the GM muscle elasticity in the proportion of red (soft tissues), green (medium tissue), and blue pixels (hard tissue) (all *p* > 0.05) (21). The second study used the strain ratio and found no significant difference in GM elasticity in the HSD group compared to the control group (*p* > 0.05), in line with the findings of the current study (22). Although not statistically significant, descriptive statistics suggested a higher proportion of soft tissues in GM muscle for JHS participants by Alsiri (21), and a higher strain ratio of the GM muscle in HSD participants in the current study (21). Notably, the mean difference in GM elasticity between the two groups was similar at 0.43 in the current study and 0.47 in the previous study (22). A type II error could explain the insignificant differences found in the current and previous studies.

To identify the most sensitive clinical examination for the biomechanical properties of HSD, the present study employed two muscular statuses: rest and maximal contraction, of different anatomical regions of the GM-AT complex. Examination at rest seems more sensitive, as it succeeded in detecting between-group differences; therefore, it is recommended for clinical examination. GM-AT musculotendinous junction, mid AT and distal AT, are recommended as anatomical regions for clinical examination. Yet, it seems that mid and distal AT are more sensitive as they showed the highest mean difference of 0.94 and 1.34 for strain ratio, respectively. Clinically, the mid AT is more applicable and standardizable being between the two malleoli. The distal AT was more difficult to examine because it is against the calcaneus. Accordingly, the current study presents three clinical examinations to determine the biomechanical properties of the GM-AT complex in HSD for patient identification, as a supplementary diagnostic tool, and for monitoring purposes. The superior test could be considered the mid-AT, followed by the distal AT, then the GM-AT musculotendinous junction, all during rest. The examination under maximum voluntary contraction was introduced to potentially increase the sensitivity of the test. It was reported previously that changing the contraction status of the AT from rest to contraction changed the strain ratio, and examination at rest showed large variabilities in strain ratio compared to contraction examination (21, 39). However, these observations were reported as a response for healthy AT (21, 39). The current study shows that for people with HSD, the examination at rest is more sensitive for detecting differences compared to contraction examinations. For contraction examination, the AT was hard to deform with the mild compressions recommended for SEG examination, therefore, a greater amount of compression was required to cause the required tissue deformation to generate elastograms. Moreover, the descriptive statistics identified by the current study could be clinically utilized to determine the expected range of strain index for patients with HSD while correlating the strain index with the color-coded images of the SEG (patients with HSD would manifest an increase in red areas of the examined structures). More importantly, recording the strain index for each patient could be potentially used as a benchmark to determine the effectiveness of management as well as the progression of the condition.

The study could be limited due to several factors. Firstly, the operator was not blinded, which could cause expectation bias. However, the SEG is equipped with a visual quality indicator to standardize the applied compressions, and the operator showed high reliability for examining the GM-AT complex (22). Patients with recent injury within the last 3 months were excluded from the study as it was considered that such structures would not accurately represent the biomechanical properties of patients with HSD due to ongoing tissue healing. However, an alternative perspective is that people with HSD may experience recurrent injuries and dislocations (11, 21), and the exclusion of such patients could have affected the generalizability of the findings be only including patients who were less severely affected by HSD. The study sample was exclusively Asian and, although ethnic differences in strain ratio values have not yet been established, care should be taken in extrapolating the findings to other populations. Shear-wave type of SEG is recommended for future research, which produces shear waves for strain measurement to remove the requirement for manual compressions applied by the examiner in compression SEG (40). Two examination statuses were used in the present study–rest and contraction–but comparing the data from these two tests is difficult because mild compressions were used at rest, and harder compressions during contraction. The current study also had a number of strengths, including a robust methodology of highly standardized procedures, focused anatomical areas, justified sample size, and in-depth statistical analysis approaches.

In conclusion, people with HSD demonstrated greater elasticity of the GM-AT complex at rest than age and gender-matched control participants. Future research is required to establish the diagnostic, clinical, and research utility of strain ratio measurement in HSD and in hEDS.

## Data availability statement

The original contributions presented in this study are included in this article/supplementary material, further inquiries can be directed to the corresponding author.

## Ethics statement

The studies involving human participants were reviewed and approved by Kuwait Ministry of Health Ethics Committee. The patients/participants provided their written informed consent to participate in this study.

## Author contributions

NA designed the study, performed the data collection and statistical analysis, and wrote the manuscript. SP designed the study, statistically analyzed part of the data, and wrote the manuscript. Both authors contributed to the article and approved the submitted version.

## Appendix

### Linear regression analysis

Linear regression equations were created considering the strain ratio as the explanatory variable with other potential predictors including age, height, weight, Beighton score, and hip, knee, and ankle pain intensity. Ethnicity was not included in the model because the sample was exclusively Asian. The strain ratio measured during rest was used in this analysis where significant differences were identified between the two groups. Three regression equations were constructed for the HSD group. However, the R square values were very low even when some of the predictors were excluded to potentially build a stronger model:

The strain ratio of the GM-AT musculotendinous junction at rest (R square 0.362, Standard Error 1.29) = (0.056) × age + (0.040) × height + (−0.042) × weight + (−0.097) × Beighton score + (−0.165) × hip pain + (0.258) × knee pain + (0.011) × ankle pain − 2.966.

The strain ratio of the mid-AT at rest (R square 0.352, Standard Error 0.352) = (0.053) × age + (0.124) × height + (0.014) × weight + (−0.200) × Beighton score + (−0.024) × hip pain + (0.305) × knee pain + (−0.222) × ankle pain − 18.311.

The strain ratio of the distal AT at rest (R square 0.259, Standard Error 2.86) = (−0.174) × age + (−0.123) × height + (0.046) × weight + (−0.119) × Beighton score + (0.509) × hip pain + (−0.453) × knee pain + (−0.124) × ankle pain + 27.433.
